# Position Estimation Based on Grid Cells and Self-Growing Self-Organizing Map

**DOI:** 10.1155/2019/3606397

**Published:** 2019-02-26

**Authors:** Baozhong Li, Yanming Liu, Hailin Li

**Affiliations:** ^1^Xidian University, Xi'an, China; ^2^College of Information and Navigation, Air Force Engineering University, Xi'an, China

## Abstract

As the basis of animals' natal homing behavior, path integration can continuously provide current position information relative to the initial position. Some neurons in freely moving animals' brains can encode current positions and surrounding environments by special firing patterns. Research studies show that neurons such as grid cells (GCs) in the hippocampus of animals' brains are related to the path integration. They might encode the coordinate of the animal's current position in the same way as the residue number system (RNS) which is based on the Chinese remainder theorem (CRT). Hence, in order to provide vehicles a bionic position estimation method, we propose a model to decode the GCs' encoding information based on the improved traditional self-organizing map (SOM), and this model makes full use of GCs' firing characteristics. The details of the model are discussed in this paper. Besides, the model is realized by computer simulation, and its performance is analyzed under different conditions. Simulation results indicate that the proposed position estimation model is effective and stable.

## 1. Introduction

Rapid development of unmanned vehicles has raised the research of autonomous navigation in recent years [1]. Getting a robust position estimation is very important for vehicles to achieve autonomous navigation tasks. Over the past 30 years, there has been much effort in solving this problem by building a map of the environment and navigating based on estimation of position in that map, the methodology of which has come to be known as simultaneous localization and mapping (SLAM) [2, 3]. Traditional approaches of SLAM are the probabilistic methods, the typical representative of which is extended Kalman filter (EKF) [4]. The computation cost of EKF is quadratic with respect to the number of the landmarks, which may result in performance degradation in large-scale environment. Many improved methods are provided to solve this problem [5], but they can only alleviate it in some extent, rather than fundamentally eliminate this problem.

Many animals exhibit perfect navigation capability in complex and large-scale environments, even when the perceived information is not exact. Taking inspiration from animals, researchers begin to propose bioinspired navigation architectures for unmanned vehicles to improve their autonomous navigation capability. Natal homing is a remarkable and common navigation behavior which enables animals such as fish, rats, and pigeons to perform long migrations to return to their natal areas or nests [6, 7]. The foundation of natal homing in animals' brains is the path integration (PI) mechanism. It can continuously accumulate self-motion information and update and provide current position vector relative to the reference position (e.g., the initial starting point) even in the absence of other external perception information such as vision [8], and this position vector provides the key navigation information for natal homing. Traditionally, PI is implemented by mathematical formulas, but the neural mechanism in animals' brains determines that PI must be realized through neural networks [9, 10]. Neural recordings from laboratory and theory research studies indicate that there are a variety of cells associated with navigation, which include place cells (PCs) [11], head direction cells (HDCs) [12, 13], grid cells (GCs) [14], and border cells (BCs) [15]. Their firing patterns are closely related to the surrounding environments and the animals' positions. Although there is no definite conclusion about how navigation behaviors are achieved in brains, it is considered that GCs are the neural basis of path integration [16].

**Figure 1:**
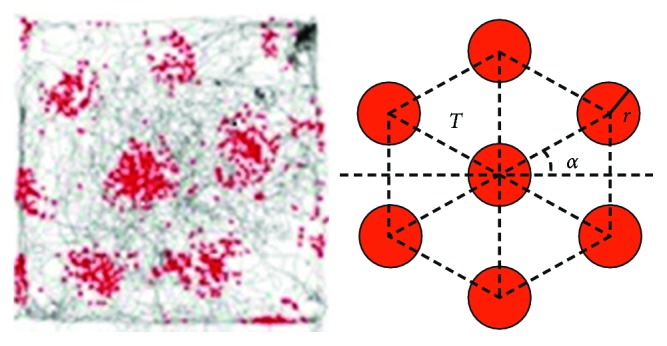
Firing pattern of a single grid cell (GC) from laboratory. (a) Firing locations and running trajectory (the gray line is the running trajectory of the rat, and each spike is plotted in red) [18]; (b) The abstract geometry of the gird fields.

GCs are a kind of neurons that exist in the entorhinal cortex (EC) of rats' brain. A GC shows multiple spatial firing fields during the movement of the animal, and these firing fields distribute in a regular hexagonal array that uniformly and periodically covers the whole environment visited by the animal [14, 17]. These spatial firing fields are independent of environmental characteristics.  Figure 1(a) shows a single GC's firing pattern from laboratory. The abstract geometry of the firing fields is also illustrated in Figure 1(b). There are mainly four parameters to characterize the firing pattern of GCs: grid period, grid orientation, firing field size, and grid phase. As presented in Figure 1(b), the grid period is defined as the median distance between the center firing field and the six surrounding firing fields. The grid orientation is defined as the angle between a fixed reference line going through the center firing field and the closest of the three main diagonals of the hexagon in counterclockwise direction. The firing field size is defined as the radius of the individual firing fields. In contrast to the above three parameters, the grid phase is not an absolute value. It describes the relative displacement between the firing fields of two co-located GCs, i.e., GCs with similar grid period, grid orientation, and firing field size. GCs that are proximate within the medial entorhinal cortex (mEC) are organized into a function module named as a GC module. The firing patterns of the cells belonging to the same GC module share the same grid period and orientation but a fixed grid phase relative to one another. The relative grid phases appear to be conserved across all environments visited by the animal. Grid periods increase discontinuously between function modules along the dorsoventral axis of mEC, with the smallest being around 25 cm and the largest so far recorded exceeding 300 cm [14, 16–19].

Currently, many types of position estimation models based on GCs have been proposed. Samsonovich and Mcnaughton [20] presented an attractor model that consists of two levels: a P-level, which is a two-dimensional sheet of neurons representing the current position of the animal, and an I-level which controls updating of the position. The position of the neural activity packet on the P-level can be continuously moved according to the input velocity. Conklin and Eliasmith [21] described a novel attractor network model which incorporates representation and updating of position into a single layer of neurons, eliminating the need for a large external control population, and without making use of multiplicative synapses. Samsonovich and Mcnaughton [20] proposed improved continuous attractor models that are capable of generating accurate regular hexagonal grid responses to the animal's position in 2-D space. Mhatre et al. [22] described a simple and general mathematical property of the trigonometry of spatial navigation and developed a neural model which was named as GRIDSmap. The model can learn to exploit the trigonometric relationship and convert path integration signals into hexagonal grid cell patterns of multiple scales, and so on. The above-mentioned models are mainly designed to simulate or reproduce the firing patterns of cells to explore the mechanism of cells' firing from the biological point of view, not for the sake of practical navigation application or improvement of navigation performance. The encoding of spatial environments and positions by navigation-related cells' firing patterns is just the first step in the process of perceived information processing. How to decode the relevant information that can be applied to practical navigation, i.e., how to establish the relationship between the cells' firing patterns and the related navigation parameters is an important subject in the research of bionic autonomous navigation.

This paper puts forward a neural network model to decode the GCs' encoding information based on the improved traditional Kohonen self-organizing map (SOM), the details of which can be found in the section of Supplementary Materials. The model makes full use of GCs' firing characteristics and can be used as an effective and stable bionic position estimation method for unmanned vehicles. Therefore, this study is beneficial to explore the neural mechanism of animals' navigation behavior as well as to provide important reference to develop bionic autonomous navigation architecture for unmanned vehicles.

The rest of this paper is organized into four sections. In Section 2, we introduce the input data model of our proposed decoding model. Then, we analyze the relationship between index vectors and positions and set up a two-level self-growing self-organizing map (SGSOM) neural network model for the estimation of positions in Section 3.  Section 4 is a simulation study of the proposed model and performance analysis. Finally, we conclude the paper in Section 5.

## 2. Data Model

A GC module is defined as a set of GCs with the same firing characteristics of grid period, grid orientation, and firing field size but a fixed grid phase relative to each other. Besides, the firing fields of all the GCs belonging to one GC module can completely cover the entire 2-D plane. Define that a GC module's period is the firing grid period of the GCs contained in the module. Hence, one GC module with a particular grid period can only encode the spatial positions periodically and ambiguously through the firing of the GCs, but GC modules of different grid periods can jointly encode the spatial positions accurately [23]. This corresponds to the well-known residue number system (RNS) which is based on the Chinese remainder theorem (CRT) [24]. The details of the CRT are described as follows.

Given *N* pairwise coprime periods *T*_1_, *T*_2_,…, *T*_*N*_ as the moduli, let *x* be a positive integer, and *R*_1_, *R*_2_,…, *R*_*N*_ be the *N* remainders of *x*, i.e.,

(1) $$\begin{array}{c} R_{i}=\mathrm{mod}\left(x,T_{i}\right), \end{array}$$

where 1 ≤ *i* ≤ *N*. Then, *x* can be uniquely reconstructed from its *N* remainders if and only if

(2) $$\begin{array}{c} 0\leq x<\text{lcm}\left\{T_{1},T_{2},\ldots ,T_{N}\right\}=\overset{N}{\underset{i=1}{\prod }}T_{i}, \end{array}$$

where lcm stands for the least common multiple of the integers. The conclusion is also established if any pair of the moduli have a common greatest common divisor (gcd) *T*. Then, the range of *x* must be limited as

(3) $$\begin{array}{c} 0\leq x<\text{lcm}\left\{T_{1},T_{2},\ldots ,T_{N}\right\}=\frac{1}{T^{N-1}}\overset{N}{\underset{i=1}{\prod }}T_{i}. \end{array}$$

**Figure 2:**
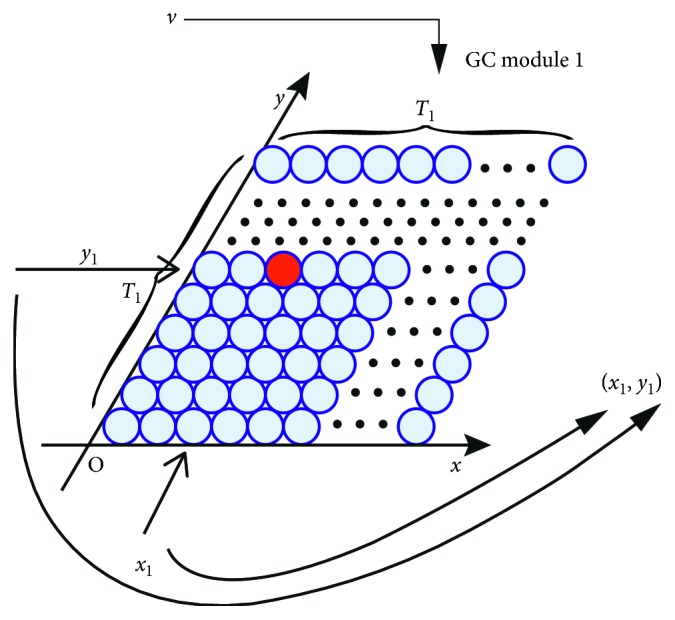
Process of generating the index coordinate in a GC module based on CAN (note that the angle of the two axes is 60 degrees). *T*_1_: period of the GC module; *v*: the vehicle's running velocity; red dot: the currently most active cell in the GC module.

**Figure 3:**
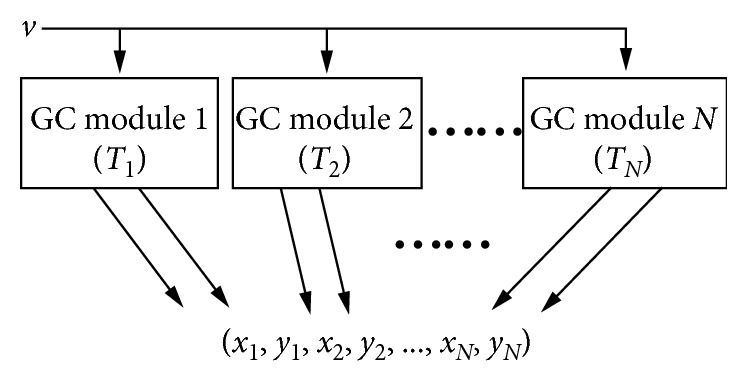
Process of generating the multiperiod GCs' firing index vector. *T*_*i*_: period of the *i*th GC module; *v*: the vehicle's running velocity; *N*: number of GC modules.

The encoding process of spatial positions using GC modules is presented in Figures 2 and 3. We apply the competitive attractor network (CAN) model to simulate the firing patterns of GCs as they have a high degree of neural plausibility [25]. In order to generate individual GCs' firing patterns, the GCs of one module need to be arranged topologically as a rhombus to form a CAN with opposite edges cyclically connected as in Figure 2 [21].

Due to the dynamic of the network, an activity packet representing the neurons' firing rates will be formed and can be moved freely by the vehicle's running velocity. When considering one axis, e.g., the *x*-axis in Figure 2, the currently most active cell's (red dot) index *x*_1_ along this axis provides the integral residue of the division of the current coordinate of the vehicle on this axis by the module's period *T*_1_. The information from one network, e.g., (*x*_1_, *y*_1_), which can be named as the index coordinate, allows to locate the vehicle periodically and ambiguously. But, according to the well-known residue number system (RNS) which is based on the Chinese remainder theorem (CRT) [24], the vector (*x*_1_, *y*_1_, *x*_2_, *y*_2_,…, *x*_*N*_, *y*_*N*_) provided by a set of *N* GC modules with *N* different periods in Figure 3, which are named as the multiperiod GCs' firing index vector (FIV), can jointly encode the spatial location accurately in a certain range [24]. Therefore, given *N* grid cell modules with *N* integer periods *T*_1_, *T*_2_,…, *T*_*N*_, which satisfy the following conditions:

(4) $$\begin{array}{c} \left\{\begin{array}{c} 0<T_{1}<T_{2}<\cdots <T_{N},\\ \text{gcd}\left(T_{i},T_{j}\right)=1,\;\left(i\neq j,1\leq i,j\leq N\right), \end{array}\right. \end{array}$$

and the radius *r* of the firing field, as illustrated in Figure 2, the number of grid cells on each side of the *N* diamond neural sheets can be calculated as

(5) $$\begin{array}{c} k_{i}=\frac{T_{i}}{2r\left(1\leq i\leq N\right)}. \end{array}$$

Obviously, if the value of *r* is properly settled, such as *r*=0.5 or 0.05 to guarantee that *k*_*i*_(1 ≤ *i* ≤ *N*) is an integer, then we can get the following result:

(6) $$\begin{array}{c} \left\{\begin{array}{c} 0<k_{1}<k_{2}<\cdots <k_{N},\\ \text{gcd}\left(k_{i},k_{j}\right)=\frac{1}{2r\left(i\neq j,1\leq i,j\leq N\right)}. \end{array}\right. \end{array}$$

According to the CRT and (3), the vector FIV=[*x*_1_, *y*_1_, *x*_2_, *y*_2_,…, *x*_*N*_, *y*_*N*_] formed by *N* pairs of index numbers from *N* grid cell modules, which we defined as the index vector, can uniquely encode the animal's current spatial position as long as the position coordinate (*x*, *y*) in the *xoy* coordinate system satisfies

(7) $$\begin{array}{c} 0\leq x,y\leq 2r\;*\text{ lcm}\left\{k_{1},k_{2},\ldots ,k_{N}\right\}\\ =2r\;*\;\overset{N}{\underset{i=1}{\prod }}\frac{k_{i}}{{\left(2r\right)}^{N-1}}. \end{array}$$

Hence, the FIV can be used as the input data model of our proposed decoding model.

## 3. Position Estimation with SGSOM

**Figure 4:**
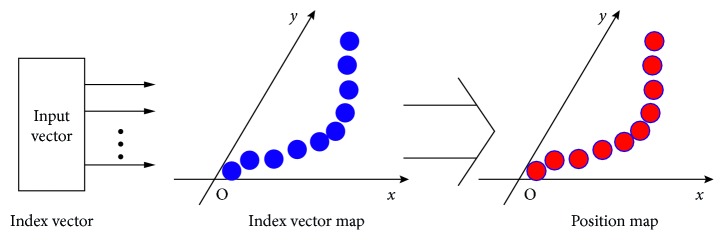
Architecture of the two-level SOM.

GC modules with different grid periods jointly encode the spatial positions based on the CRT. Theoretically, the decoding of the information involves complex multiplications and modular inversion operations [24], which make it difficult to be implemented through neural network. Hence, based on the properties of the RNS and the characteristics of SOM, we propose a neural network model to estimate the current position of the vehicle according to the input FIV. The main structure of our model is shown in Figure 4. It consists of a two-level SGSOM neural network. The first-level SGSOM is an ordering process, and the topological order of input vectors is reproduced in the two-dimensional space. The second-level SGSOM is a 1-1 mapping process from one two-dimensional space to another two-dimensional space. Each cell of the second map represents a position. The details of each level will be discussed in the following sections.

### 3.1. First-Level SGSOM

The real-time requirement of position estimation determines that there are no sample vectors for the training of SOM when the vehicle begins to explore a new environment. Thus, the weight vectors of competitive layer neurons should be provided in advance rather than through training. This is also neural plausibility as many cells' firing patterns become stable when animals reach adult ages [16]. In addition, a fixed number of neurons for position estimation cannot adapt to scale changes of the environments. Therefore, we propose a SGSOM based on the characteristics of traditional SOM in our model. In contrast to traditional SOM, the number of its competitive layer neurons is not fixed. New neurons can be produced and added to the competitive layer of the SGSOM with the exploration of the environment, and the weight vectors of the newly added neurons are generated by prediction of the exploring trajectory. The network working steps are as follows.

**Step 1 .**

Initialize the network.

Generate the first neuron of the competitive layer and take the first input FIV as its weight vector:

(8) $$\begin{array}{c} W_{1}={\text{FIV}}_{1}, \end{array}$$

where *W*_1_=[*u*_1_^1^, *v*_1_^1^, *u*_2_^1^, *v*_2_^1^,…, *u*_*N*_^1^, *v*_*N*_^1^] is the weight vector of the first competitive layer neuron and FIV_1_=[*x*_1_^1^, *y*_1_^1^, *x*_2_^1^, *y*_2_^1^,…, *x*_*N*_^1^, *y*_*N*_^1^] is the first input index vector.

**Step 2 .**

Calculate the Euclidean distance between the input vector and each competitive layer neuron's weight vector, and identify the winning neuron which has the minimum distance.

Firstly, we introduce some properties of the RNS. Consider that *x* and *y* are two arbitrary integers and *m* and *n* are two positive integers as moduli with *m* < *n*, then

(9) $$\begin{array}{c} \left\{\begin{array}{c} r_{x}^{m}=mod\left(x,m\right),\\ r_{x}^{n}=mod\left(x,n\right), \end{array}\right.\\ \left\{\begin{array}{c} r_{y}^{m}=mod\left(y,m\right),\\ r_{y}^{n}=mod\left(y,n\right). \end{array}\right. \end{array}$$

According to the relative properties of the RNS, the following relationship is established:

(10) $$\begin{array}{c} {\langle x-y\rangle }_{\left\{m,n\right\}}=\left\{r^{m},r^{n}\right\}\\ =\left\{<r_{x}^{m}-r_{y}^{m}{>}_{m},<r_{x}^{n}-r_{y}^{n}{>}_{n}\right\}, \end{array}$$

where 〈*x* − *y*_{*m*, *n*}_〉={mod(*x* − *y*, *m*), mod(*x* − *y*, *n*)} and 〈*x*〉_*m*_=mod(*x*, *m*). Suppose *d*=*x* − *y*. We can get the following conclusion:

(11) $$\begin{array}{c} \left\{\begin{array}{cc} r^{m}=r^{n}=d, & \text{if}\;\;0\leq d<m,\\ m-r^{m}=n-r^{n}=-d, & \text{if}\;\;-m<d<0. \end{array}\right. \end{array}$$

These conclusions are still established when there are more than two moduli. Integers *x* and *y* can also be extended to multidimensional vectors, each dimension of which can be treated independently.

Assume that there are *M* neurons in the competitive layer with weight vector as *W*_*i*_(1 ≤ *i* ≤ *M*) and FIV=[*x*_1_, *y*_1_, *x*_2_, *y*_2_,…, *x*_*N*_, *y*_*N*_] is the current input index vector. According to the above conclusions, we can have

(12) $$\begin{array}{c} {\langle \text{FIV}-W_{i}\rangle }_{\left\{k_{1},k_{2},\ldots ,k_{N}\right\}}=\left\{d_{1}^{x},d_{1}^{y},d_{2}^{x},d_{2}^{y},\ldots ,d_{N}^{x},d_{N}^{y}\right\}\\ =\left\{{\langle x_{1}-u_{1}^{i}\rangle }_{k_{1}},{\langle y_{1}-v_{1}^{i}\rangle }_{k_{1}}{\langle x_{2}-u_{2}^{i}\rangle }_{k_{2}},{\langle y_{2}-v_{2}^{i}\rangle }_{k_{2}}\ldots {\langle x_{N}-u_{N}^{i}\rangle }_{k_{N}},{\langle y_{N}-v_{N}^{i}\rangle }_{k_{N}}\right\}. \end{array}$$

According to the geometric relationship and the cosine theorem, the Euclidean distance between FIV and *W*_*i*_(1 ≤ *i* ≤ *M*) can be calculated as

(13) $$\begin{array}{c} \text{dis}\left(\text{FIV},W_{i}\right)=\sqrt{{\left(d^{x}\right)}^{2}+{\left(d^{y}\right)}^{2}-2\;*\;d^{x}\;*\;d^{y}\;*\;\cos\left(\frac{2\pi }{3}\right)}, \end{array}$$

(14) $$\begin{array}{c} \left\{\begin{array}{cc} d^{j}=d_{1}^{j}, & \text{if}\;\;d_{i}^{j}=d_{i^{\prime }}^{j},\\ d^{j}=T_{1}-d_{1}^{x}, & \text{if}\;\;T_{i}-d_{i}^{j}=T_{i^{\prime }}-d_{i^{\prime }}^{j},\\ d^{j}=+\infty , & \text{other cases}, \end{array}\right. \end{array}$$

where *i*, *i*′=1,2,…, *N*; *j*=*x*, *y*.

**Figure 5:**
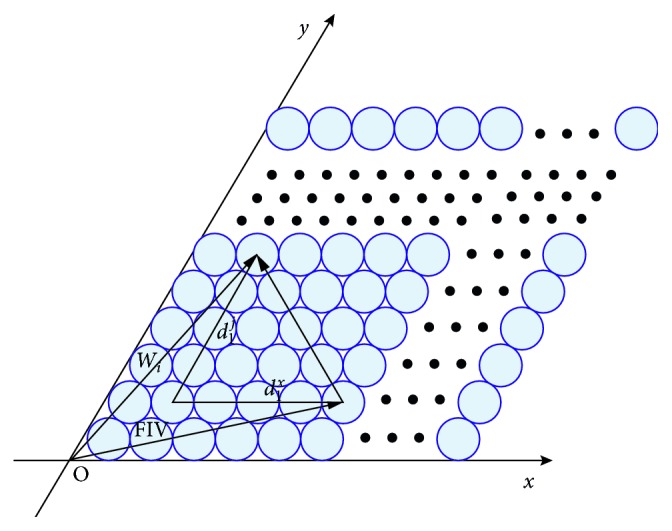
Calculation process of Euclidean distance of one case.

Figure 5 presents the calculation process of case 1. It is obvious that the less the Euclidean distance is, the closer the two vectors are, so does the two positions contained in the two vectors. The neuron which has the minimum Euclidean distance with FIV is considered as the winning neuron.

**Figure 6:**
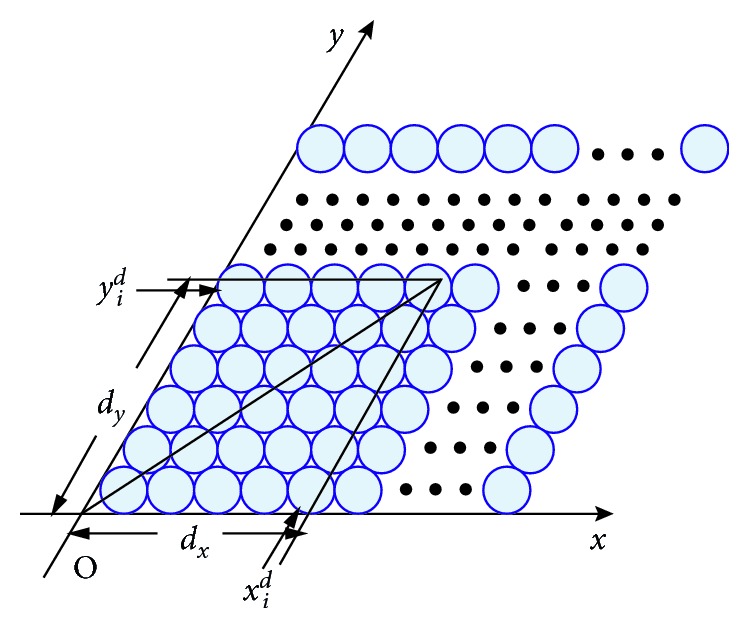
Displacement during a sampling interval and its transformation to a index vector.

Suppose that the current perceived velocity is *v*(*t*), the running direction is *θ*(*t*), and they remain unchanged during the next sampling interval Δ*t*. Then, the displacement components along two axes of the *xoy* coordinate system during Δ*t* can be predicted as

(15) $$\begin{array}{c} \left\{\begin{array}{c} d_{x}=v\left(t\right)\;*\;\Delta t\;*\;\cos\left(\theta \left(t\right)\right)-v\left(t\right)\;*\;\Delta t\;*\;\frac{\sin\left(\theta \left(t\right)\right)}{\tan\left(\pi /3\right)},\\ d_{y}=v\left(t\right)\;*\;\Delta t\;*\;\frac{\sin\left(\theta \left(t\right)\right)}{\sin\left(\pi /3\right)}, \end{array}\right. \end{array}$$

which are illustrated in Figure 6. In order to guarantee that there must be a winning neuron in this step, according to (11), the following expression must be satisfied:

(16) $$\begin{array}{c} \left\{\begin{array}{c} \text{abs}\left(d_{x}\right)<2r\;*\;k_{1},\\ \text{abs}\left(d_{y}\right)<2r\;*\;k_{1}, \end{array}\right. \end{array}$$

where abs(*d*) means to take the absolute value of *d*.

**Step 3 .**

Predict a weight vector and add a new neuron to the competition layer.

The displacement presented in (15) can be transformed into an index vector based on the *N* grid cell modules as illustrated in Figure 6. Thus, we can get the displacement index vector as

(17) $$\begin{array}{c} v_{d}=\left\{x_{1}^{d},y_{1}^{d},x_{2}^{d},y_{2}^{d},\ldots ,x_{N}^{d},y_{N}^{d}\right\}, \end{array}$$

where

(18) $$\begin{array}{c} \left\{\begin{array}{c} x_{i}^{d}=\text{round}\left(\frac{\mathrm{mod}\left(d_{x},k_{i}\right)}{\left(2r\right)}\right),\\ y_{i}^{d}=\text{round}\left(\frac{\mathrm{mod}\left(d_{y},k_{i}\right)}{\left(2r\right)}\right), \end{array}\right. \end{array}$$

where *i*=1,2,…, *N*, and round(*x*) means to take the integer nearest *x*. Suppose that there are *M* neurons in the competitive layer, the *j*th neuron is the current winning neuron, and its weight vector is *W*_*j*_=[*u*_1_^*j*^, *v*_1_^*j*^, *u*_2_^*j*^, *v*_2_^*j*^,…, *u*_*N*_^*j*^, *v*_*N*_^*j*^], then the predicted weight vector of the neuron that may be added to the competitive layer can be written as

(19) $$\begin{array}{c} W=\left\{u_{1},v_{1},u_{2},v_{2},\ldots ,u_{K},v_{K}\right\}, \end{array}$$

where

(20) $$\begin{array}{c} \left\{\begin{array}{cc} u_{i}=\mathrm{mod}\left(x_{i}^{d}+u_{i}^{j},k_{i}\right), & \left(i=1,2,\ldots ,N\right),\\ v_{i}=\mathrm{mod}\left(y_{i}^{d}+v_{i}^{j},k_{i}\right), & \left(i=1,2,\ldots ,N\right). \end{array}\right. \end{array}$$

Check whether or not *W* exists in the weight vectors of the *M* competitive layer neurons. If the answer is yes, turn to step 2. Otherwise, add a new neuron to the competitive layer and set its weight vector as *W* and then turn to step 2.

**Step 4 .**

Repeat Steps 2 and 3 until the exploration of the environment is completed.

### 3.2. Second-Level SGSOM

The second-level SGSOM is a 1-1 mapping from the first level. They have the same number of neurons. Each neuron in this level represents a position, which is obtained according to the additions of the first-level neurons. The network working steps are as follows.

**Step 5 .**

Initialize the Network.

Generate the first neuron of this level, set its contained position coordinate as (0,0) or (*x*_0_′, *y*_0_′) in the Cartesian coordinate system, and associate it with the first neuron of the first level.

**Step 6 .**

Output the estimated position coordinate.

For any input index vector, output the position coordinate contained in the neuron which is associated with the winning neuron of the first level. This position coordinate can be directly used as the estimation of the vehicle's current position.

**Step 7 .**

Add a new neuron to this level.

If a new neuron is just added to the first level, then a new neuron should also be added to this level and associated with the newly added neuron of the first level. Suppose that a new neuron has been just added to the first level and second level, respectively, the *j*th neuron is the current winning neuron of the first level, and its associated neuron of the second level contains position coordinate (*x*_*j*_′, *y*_*j*_′). According to the predicted index vector of the displacement shown in (17), the position coordinate (*x*′, *y*′) contained in the second level's newly added neuron can be written as

(21) $$\begin{array}{c} \left\{\begin{array}{c} x^{\prime }=x_{j}^{\prime }+\Delta x^{\prime },\\ y^{\prime }=y_{j}^{\prime }+\Delta y^{\prime }, \end{array}\right. \end{array}$$

where Δ*x*′ and Δ*y*′ are calculated as

(22) $$\begin{array}{c} \left\{\begin{array}{c} \Delta x^{\prime }=\left(d^{x}+d^{y}\;*\text{ cos}\left(\frac{\pi }{3}\right)\right)\;*\;r,\\ \Delta y^{\prime }=d^{y}\;*\text{ sin}\left(\frac{\pi }{3}\right)\;*\;r, \end{array}\right. \end{array}$$

where *d* ^*x*^ and *d* ^*y*^ are calculated as

(23) $$\begin{array}{c} \left\{\begin{array}{cc} d^{j}=d_{1}^{j}, & \text{if}\;\;d_{i}^{j}=d_{i^{\prime }}^{j},\\ d^{j}=d_{1}^{x}-T_{1}, & \text{if}\;\;d_{i}^{j}-T_{i}=d_{i^{\prime }}^{j}-T_{i^{\prime }}, \end{array}\right. \end{array}$$

where *i*, *i*′=1,2,…, *N*; *j*=*x*, *y*.

## 4. Results and Analysis

In our model, the relationship between the firing of GCs and the vehicle's position is discussed, and we mainly focus on the position estimation performance that the proposed model can decode from GCs' firing patterns. Hence, the analysis of this model is based on the comparison between the position information encoded by GCs' firing patterns and the position information decoded by the proposed model. Simulations are divided into two parts. First, position estimations are carried in linear motion and curvilinear motion. Second, the influence of model parameters is analyzed.

### 4.1. Realization of the Model

In this section, simulations are carried out to verify the effectiveness and stability of the proposed scheme. The simulation parameters are listed in Table 1. The performance of the model is evaluated by the position estimation error (PEE) which is defined as follows:

(24) $$\begin{array}{c} \text{PEE}=\sqrt{{\left(x^{\prime }-{\hat{x}}^{\prime }\right)}^{2}+{\left(y^{\prime }-{\hat{y}}^{\prime }\right)}^{2}}, \end{array}$$

where (*x*′, *y*′) and $\left({\hat{x}}^{\prime },{\hat{y}}^{\prime }\right)$ are the real and estimated position coordinates of the vehicle in the rectangular Cartesian coordinate system. According to (15) and (16), in order to guarantee that the proposed model can work properly, the velocity should be limited by the following formula:

(25) $$\begin{array}{c} v\;*\;\Delta t<2r\;*\;k_{1}=67\left(\text{m}\right). \end{array}$$

In other words, the value of velocity *v* should be less than 134 m/s.

**Figure 7:**
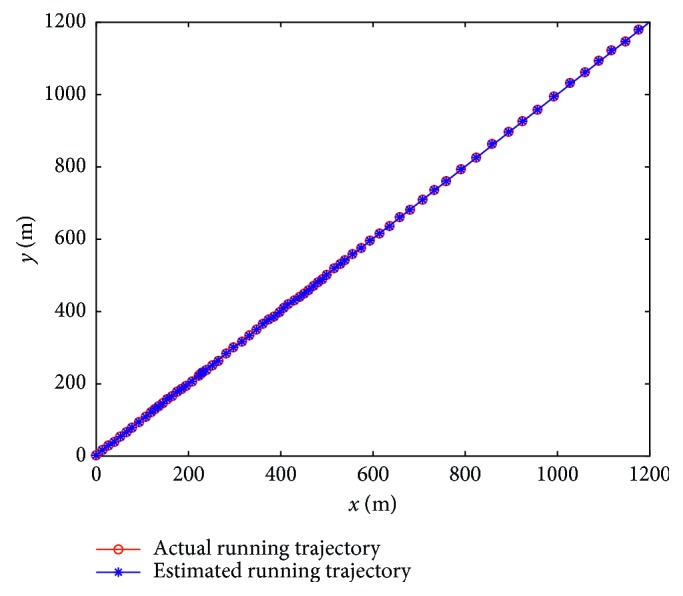
Actual running trajectory and estimated running trajectory in linear motion.

**Figure 8:**
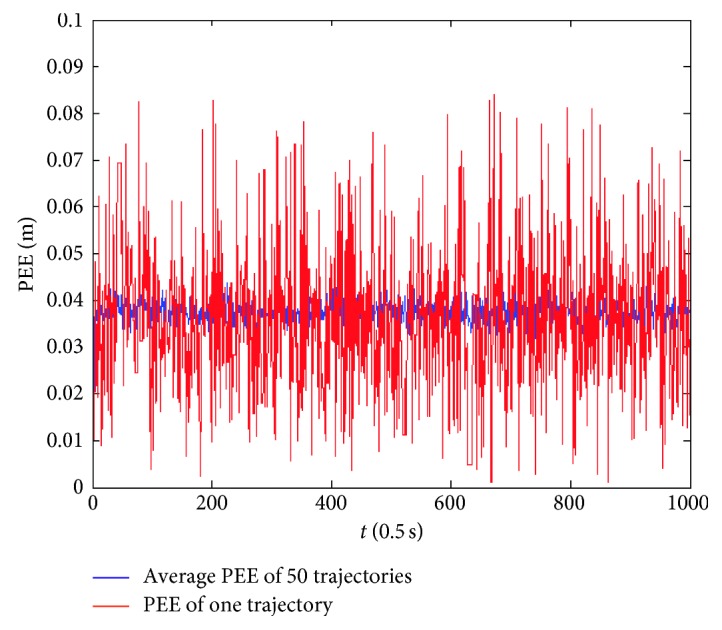
Average PEEs of 50 trajectories and PEEs of one trajectory in linear motion.

**Figure 9:**
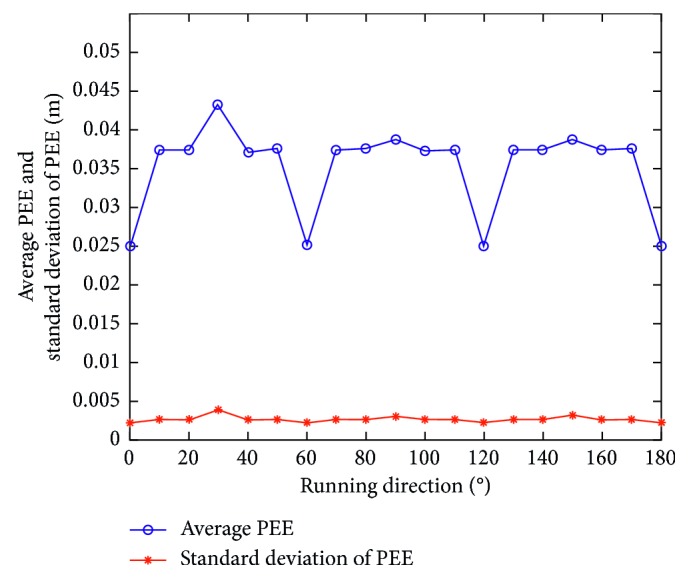
Average PEEs and standard deviations of PEEs of different running directions in linear motion.

**Figure 10:**
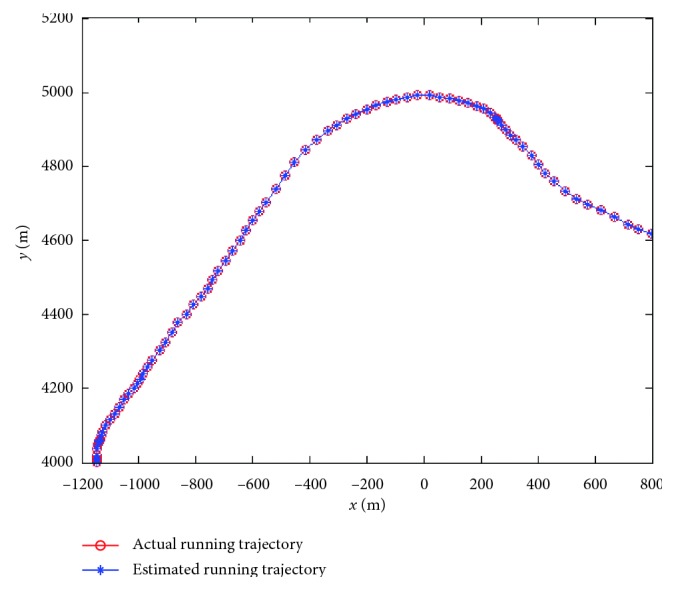
Actual running trajectory and estimated running trajectory in curvilinear motion.

**Figure 11:**
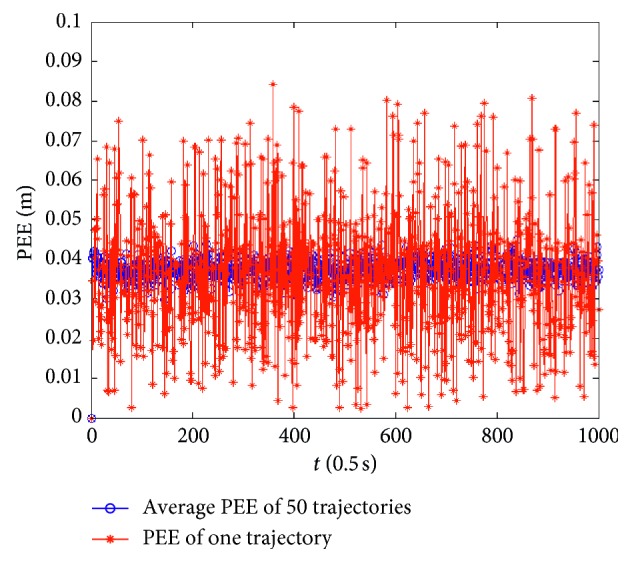
Average PEEs of 50 trajectories and PEEs of one trajectory in curvilinear motion.

**Figure 12:**
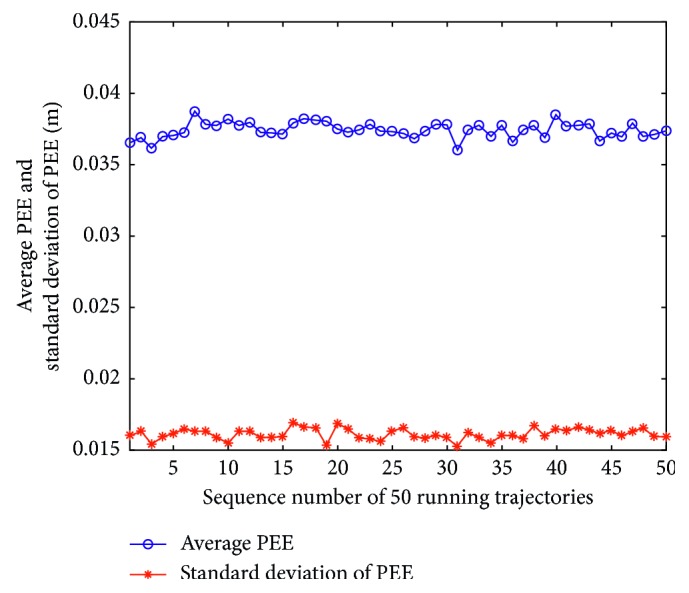
Average PEEs and standard deviations of PEEs of different trajectories in curvilinear motion.

Firstly, assume that the perceived velocity and running direction are exact and remain unchanged during a sampling interval and the running direction also remains unchanged during the total running time.  Figure 7 presents an actual running trajectory and a trajectory formed by the estimated positions in linear motion. The running direction is taken as 45° relative to the *x*-axis. As illustrated, the two trajectories almost overlap each other, which indicates that the proposed model can decode the position information contained in the index vectors very well.  Figure 8 presents the PEE of one trajectory and average PEE of 50 trajectories in a fixed running direction.  Figure 9 presents the results when the vehicle runs in different directions. In this simulation, the vehicle runs along 50 trajectories with random velocity *v* which is less than 74 m/s in each direction. The results presented in Figure 9 are obtained by first averaging the PEEs of all the sampled positions for each trajectory and then averaging all the obtained average PEEs of 50 trajectories in each direction. The maximum of average PEEs is less than 0.045 m. The difference of average PEEs between different running directions is smaller than 0.02 m. The standard deviations of PEEs in different running directions are also offered based on these average PEEs, which is less than 0.005 m. Thus, these simulation results indicate that the proposed model is effective and stable in the case of linear motion. Simulations are also carried out when the vehicle runs randomly in a 2-D environment.  Figure 10 presents a real running trajectory and its corresponding estimated running trajectory. The two trajectories also almost overlap each other, which indicates the proposed model can also decode the position information contained in the curvilinear motion.  Figure 11 presents the PEE of one trajectory and average PEE of 50 random trajectories. The average PEEs and standard deviation of PEEs are also calculated for each trajectory in Figure 12. The difference of PEEs among different trajectories is still very small, which suggests that the performance of the proposed position estimation model is stable.

To sum up, the proposed position estimation model based on the two-level SGSOM is effective and stable.

### 4.2. Influence of Model Parameters on Position Estimation

**Figure 13:**
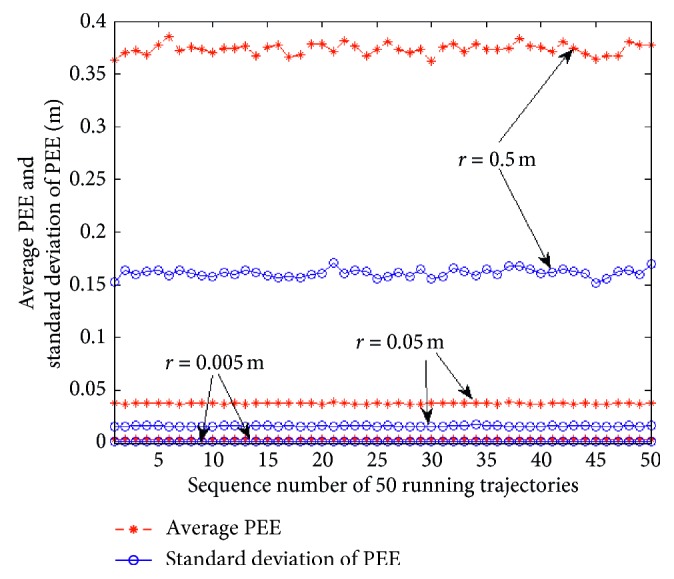
Average PEEs and standard deviations of PEEs of different firing field radii in curvilinear motion.

The parameters related to position estimation results mainly include the number of grid cell modules, the grid periods of the grid cell modules, and the radius of the firing field. The sizes of grid cell modules depend on the above three parameters. Given *N* grid cell modules, there are *N* grid periods *T*_1_, *T*_2_,…, *T*_*N*_. According to the CRT and (2), these three parameters determine the range of the explored environment within which our proposed model can work well. The greater these three parameters are, the larger the range of the explored environment is. In addition, according to (13) and (14), the minimum period *T*_1_ as well as *k*_1_ determines the max. velocity of the vehicle. The radius of grid cells' firing field can not only affect the range of the environment but also affect the PEE of the model.  Figure 13 presents the simulation results according to three different radii of GCs' firing fields. The results show that the smaller the radius of grid cells' firing field, the smaller the average PEE and standard deviation of PEE are. But, it is obvious that the computation complexity of the model become larger with the decrease of the radius.

## 5. Conclusions and Discussion

In this paper, a scheme based on the firing patterns of GCs and SGSOM is set up for the estimation of positions. By using the topology preserving of SOM, we set up a map from firing index vectors to positions through SOM, which maps similar index vectors into adjacent positions. To illustrate the performance of the scheme, simulation experiments are done to test the model and the results indicate its effectiveness and stability. Furthermore, the influence of model parameters on position estimation is also analyzed.

In this model, there is no need to carry out the complex multiplications and modular inversion operations like the solution of the CRT. When the vehicle arrives at a new place, just put the index vector generated by the grid cell models into the network, then the estimation of position coordinate can be obtained. Moreover, the size of SGSOM is changeable with the scales of the environments, which is very useful for conserving computation resource. In conclusion, the use of SGSOM network on position estimation is worthy of being applied in practice. Decades of research on the neurobiology of navigation focused on two-dimensional (2-D) navigation on flat surfaces, which laid the foundations for understanding the neural basis of 2-D spatial navigation. Thus, our research is also carried out in a 2-D environment. It is obvious that the results of our research can be directly extended to three-dimensional (3-D) environment.

In this paper, we mainly focus on the position estimation performance of the proposed model from the viewpoint of bionic navigation. As a decoder, our model can accurately restore the position information contained in the index vectors, and the estimation error can be neglected as for navigation. Therefore, we do not compare its performance with that of other different models of position estimation. Besides, considering that the perceived speed is not always exact due to kinds of factors such as noise in practical application, the position information contained in the index vectors and the decoded position coordinates will not be accurate compared with vehicles' real positions, and moreover, the errors will be accumulated in the future. This is a common and remarkable problem in the field of position estimation based on self-motion information. The main solutions for this problem are multisource information fusion methods such as loop detection based on vision information [26]. We will make further study on this topic in the next research work.

## Figures and Tables

**Table 1:** Simulation parameters.

Parameter	Value
Number of GC modules	3
Periods of GC modules	67 m, 68 m, 69 m
Radius of GCs' firing fields	0.05 m
Sizes of GC modules	670 *∗* 670,680 *∗* 680,690 *∗* 690
Sampling interval	0.5 s
Total running time	500 s

## Data Availability

The data used to support the findings of this study are included within the article.

## Supplementary Materials

Supplementary Materials The self-organizing map introduced in this paper is a Kohonen self-organizing map, which is also named as Kohonen feature map [27]. It is an unsupervised, competitive feed-forward neural network. SOM can map high-dimensional data into one- or two-dimensional data keeping the same topological order as the original data, and then features of the input data will be visualized. Thus, SOM is an effective method for feature extraction and dimensionality reduction and usually used for classification or prediction of different problems. Traditional SOM network consists of two layers: input layer and competitive layer which is also called output layer. The number of input layer nodes is equal to the input vector's dimension. The number of competitive layer neurons is fixed, and the neurons are usually arranged in a two-dimensional rectangle or hexagon grid. Each neuron of the competitive layer contains a weight vector that has the same dimension as the input vector. The whole network works as follows. When an input sample vector is put into the network, the Euclidean distance between this vector and weight vectors of the competitive layer neurons is calculated to obtain the winning neuron which has the minimum distance. Then, the weight vectors of the winning neuron and its neighboring neurons are adjusted to make them more similar to the input sample vector. Eventually, the weight vectors of competitive layer neurons have a certain distribution as input sample vectors by such training.
